# Newborn Screening in a Pandemic—Lessons Learned

**DOI:** 10.3390/ijns9020021

**Published:** 2023-04-11

**Authors:** Matej Mlinaric, James R. Bonham, Viktor Kožich, Stefan Kölker, Ondrej Majek, Tadej Battelino, Ana Drole Torkar, Vanesa Koracin, Dasa Perko, Ziga Iztok Remec, Barbka Repic Lampret, Maurizio Scarpa, Peter C. J. I. Schielen, Rolf H. Zetterström, Urh Groselj

**Affiliations:** 1 Department of Endocrinology, Diabetes and Metabolic Diseases, University Children’s Hospital, UMC Ljubljana, Bohoričeva Ulica 20, 1000 Ljubljana, Slovenia; 2 Faculty of Medicine, University of Ljubljana, Vrazov Trg 2, 1000 Ljubljana, Slovenia; 3 Office of the International Society for Neonatal Screening, Reigerskamp 273, 3607 HP Maarssen, The Netherlands; 4 Sheffield Children’s NHS Foundation Trust, Western Bank, Sheffield S10 2TH, UK; 5 Department of Pediatrics and Inherited Metabolic Disorders, Charles University-First Faculty of Medicine, and General University Hospital in Prague, Ke Karlovu 455/2, 128 08 Praha, Czech Republic; 6 Division of Child Neurology and Metabolic Medicine, Center for Child and Adolescent Medicine, Heidelberg University Hospital, Im Neuenheimer Feld 430, 69120 Heidelberg, Germany; 7 National Screening Centre, Institute of Health Information & Statistics of the Czech Republic, 128 01 Prague, Czech Republic; 8 Department of Dermatovenerology, General Hospital Novo Mesto, 8000 Novo Mesto, Slovenia; 9 Clinical Institute for Special Laboratory Diagnostics, University Children’s Hospital, UMC Ljubljana, 1000 Ljubljana, Slovenia; 10 Regional Coordinating Center for Rare Diseases, European Reference Network for Hereditary Metabolic Diseases (MetabERN), Udine University Hospital, Piazzale Santa Maria Della Misericordia 15, 33100 Udine, Italy; 11 Center for Inherited Metabolic Diseases, Karolinska University Hospital, 141 86 Stockholm, Sweden; 12 Department of Molecular Medicine and Surgery, Karolinska Institutet, 141 86 Stockholm, Sweden

**Keywords:** newborn screening, COVID-19, NBS, contingency plan, pandemic, telemedicine, dried blood spot

## Abstract

The COVID-19 pandemic affected many essential aspects of public health, including newborn screening programs (NBS). Centers reported missing cases of inherited metabolic disease as a consequence of decreased diagnostic process quality during the pandemic. A number of problems emerged at the start of the pandemic, but from the beginning, solutions began to be proposed and implemented. Contingency plans were arranged, and these are reviewed and described in this article. Staff shortage emerged as an important issue, and as a result, new work schedules had to be implemented. The importance of personal protective equipment and social distancing also helped avoid disruption. Staff became stressed, and this needed to be addressed. The timeframe for collecting bloodspot samples was adapted in some cases, requiring reference ranges to be modified. A shortage of essential supplies and protective equipment was evident, and laboratories described sharing resources in some situations. The courier system had to be adapted to make timely and safe transport possible. Telemedicine became an essential tool to enable communication with patients, parents, and medical staff. Despite these difficulties, with adaptations and modifications, some centers evaluated candidate conditions, continued developments, or began new NBS. The pandemic can be regarded as a stress test of the NBS under real-world conditions, highlighting critical aspects of this multidisciplinary system and the need for establishing local, national, and global strategies to improve its robustness and reliability in times of shortage and overloaded national healthcare systems.

## 1. Background

Newborn screening (NBS) is an essential component of public health, as it enables the early detection, diagnosis, and treatment of numerous disorders of metabolism and other diseases in as yet asymptomatic newborns, preventing irreversible damage or even death and enabling normal development and growth [1,2]. Notably, large international and regional differences exist regarding the organization and scope of NBS [3,4,5]. 

The NBS testing pathway includes the collection of the dried blood sample (DBS) at a given time frame (often 48 h to 72 h) after the birth of a baby; transportation of the sample to the laboratory; testing the sample; requests for a new sample test if necessary; reporting of normal results (screen negative) or communicating the abnormal (screen positive) results; referral of children with abnormal results to appropriate clinical specialists; and life-long follow-up of confirmed cases [4,6]. The continuous education of laypersons and professionals is also an important part of screening processes [7,8]. Screening should also be monitored regularly to ensure the efficacy of the program, and from time to time, new disorders need to be considered and introduced [9]. A disruption of any step can have serious consequences for newborns [6].

The COVID-19 pandemic has had a profound effect on healthcare systems, including NBS services [10]. A decrease in the diagnosis of inherited metabolic disorders was reported, and missed cases are suspected in some centers [10,11]. On the other hand, some centers did not report any missed cases of inherited metabolic disorders by NBS [10]. The health risks of individuals with rare diseases, such as inherited metabolic diseases, were mostly increased by problems with medical supply, less frequent visits to metabolic centers, and an overall reduction in medical services but less by SARS-CoV-2 itself, with some exceptions [12,13]. Healthcare staff working within NBS became ill, and staff shortages were reported as a result. Shipping and courier services experienced delays. Outpatient clinic visits were reduced, and families were reluctant to visit the hospital. Information technology became of major importance, and the use of telemedicine emerged and increased as a result [10]. Care for diagnosed patients was not optimal, and therapy was disrupted during the COVID-19 pandemic [14].

Incident reporting, identifying weaknesses, and constantly seeking solutions through the cooperation of different parts of NBS services should be an integral part of the NBS system [15]. We report experiences during the COVID-19 pandemic and aim to identify examples of good practices that could prove valuable during future pandemics or in other public health emergencies.

## 2. Methods

We searched the PubMed^®^ (National Library of Medicine, Washington, DC, USA) database using the MeSH terms “newborn screening” and “COVID-19” for appropriate reports written in English. A total of 344 articles were found with those search terms, and all titles were reviewed. All articles that were not about laboratory newborn screening during the pandemic were excluded. In ambiguous titles, the abstract was red. A total of 14 articles were included in the end. At the second stage, we checked the titles of the first 100 articles found with Google Scholar^®^ (Alphabet Inc., Los Angeles, CA, USA) using the search term “newborn screening” and “COVID-19”; by repeating the process used in PubMed^®^ (National Library of Medicine, Washington, DC, USA), an additional 2 articles were found. Furthermore, we reviewed the citations made in these articles to find additional articles and webpages describing problems and solutions encountered during the COVID-19 pandemic or already present contingency plans (13 articles or pages). In the main text, we grouped the problems and advices into 10 categories: staff working on NBS (Section 3), newborns and their family (Section 4), collecting samples (Section 5), transportation (Section 6), supplies and technical assistance (Section 7), laboratory evaluation (Section 8), communication (Section 9), management of abnormal screening results (Section 10), hospitals and maternal wards (Section 11), and implementation of novel screening methods (Section 12).

**Figure 1:**
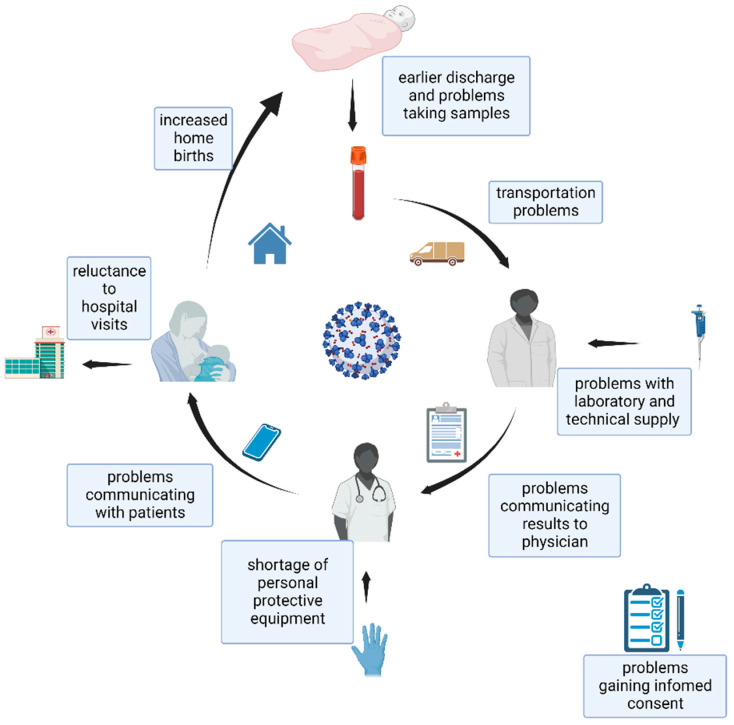
Problems seen during the COVID-19 pandemic in newborn screening (created with BioRender.com (BioRender, Toronto, ON, Canada)).

In the first part of the text, the problems found are mentioned under the headers (Figure 1), and in the second part, the advice and good practices used during the COVID-19 pandemic are presented. For problems that were not sufficiently mentioned in the articles directed to newborn screening, we searched articles for advice from other related fields of medicine (maternal care, inherited metabolic disorders, etc.). 

## 3. Challenges Faced by Staff Working on Newborn Screening Programs

The COVID-19 pandemic was a stressful period for the staff working within NBS. Staff employed within essential aspects of the pathway (laboratories, hospitals, and couriers) faced shortages due to sickness, quarantine, and school and daycare closures, or the diversion of staff and resources to support activities directly related to meeting the needs created by COVID-19. Staff shortage and changes in routine led to additional work and increased stress, anxiety, and burnout among the healthcare workforce [10,15,16]. The additional workload often prevented the use of annual leave, and measures to allow this to be deferred were implemented [16].

Physical distancing and the usage of personal protective equipment were recommended to reduce additional COVID-19 infections in the NBS teams [15]. Monitoring to identify pyrexia and other signs of possible COVID-19 were undertaken, and if positive signs were present or contact with a COVID-19-positive person was established, testing for SARS-CoV-2 was performed. Established work patterns and workflows were also modified [16]. In Texas, a six-day work week was implemented to minimize delays in testing [15]. Working in two shifts was introduced in some centers (for example Victoria, Australia, and Prague, Czech Republic), alternating the workforce on a weekly or 3-day basis to reduce the risk of COVID-19 infections spreading among the whole team [6,10]. If possible, data entry and follow-up work was performed remotely to reduce the number of staff together at the workplace. The opening of specimens and mail was performed at a location separate from the laboratory [16]. Additional staff had to be trained rapidly to undertake essential tasks, and those already working had to learn additional skills to support more flexible working patterns [16,17,18]. Measures to recognize and prevent burnout were introduced to cope with the increased workload and reduce its burden on the staff [16]. Strategies to promote well-being were introduced, and some NBS team members completed training as Peer Support workers [19]. In some places, NBS was designated as an essential service, reducing the relocation of staff in the laboratory to support COVID-19 testing [16].

## 4. Challenges Faced in the Management of Newborns and Families Participating in Newborn Screening

There was an increase in searches for homebirths and an actual increase in number of homebirths during the COVID-19 pandemic [20,21]. Patients also became reluctant to revisit the hospital for NBS testing [16]. Patients faced problems getting to the NBS facilities for blood draws because of restrictions on provincial borders [22]. The education of midwives was required using on-line training material, so that newborns could be tested in a home setting or sent to the hospital [15]. To cross the regional borders, ambulances were sometimes used to transport the patients [22].

## 5. Challenges Faced in Collecting Samples for Newborn Screening

In some countries, parents have to give informed consent for NBS, while in others, participation is mandatory [4]. In normal conditions, the NBS specimen has to be taken at a specific day of life for which reference ranges are determined [16]. During the COVID-19 pandemic, the duration of hospitalization for mothers and newborns was reduced to minimize patients’ stays [9,23]. To attempt to maintain the sensitivity and specificity of testing, revised reference ranges were determined in some centers. Many followed the moto it is better to screen early than not at all [6,7,16]. Additional non-NBS staff and nurses had to be trained to undertake NBS sampling to substitute for missing personnel and reduce staff shortages. There were reports that the use of newly trained and less experienced staff resulted in blood spot samples of poorer quality [15]. The Indiana state department of health re-educated the personnel on the DBS collection procedure to minimize the need for unnecessary repeated sampling [7]. If DBS collection was still not optimal, recollection before discharge was suggested. The service in Missouri recommended that families stay at the hospital for at least 24 h to minimize recollection due to an early sample [7]. Suggestions for gaining informed consent are described under the research headings.

## 6. Challenges Faced in Transportation

The samples collected at the maternity ward have to be transported to the laboratory. When the distance is short, carriers or tube systems could be used. To traverse longer distances from the maternity ward to the laboratory, couriers become important [16]. Stricter border controls between countries or even between provinces made it difficult to transport medical foods, supplies, and medicines [22]. 

Couriers themselves were also susceptible to COVID-19 infections, resulting in staff shortages. Additional workload was also evident because of a general increase in shipments by courier [16]. In some hospitals, couriers were not allowed into the hospital, so a hospital staff member had to be assigned to meet the courier and receive the NBS samples. In more problematic sites, “lockboxes” were employed [7]. The courier services frequently had to adopt new strategies to sustain the delivery and collection of NBS specimens in a reasonable time frame [16]. To reduce contact and the spread of COVID-19, the number of deliveries was reduced by changing inventory practices and by ordering extra stocks of essential consumables [17,18].

To pass the border controls, NBS specimens in the Philippines had, in some cases, to be transported to the provincial border and picked up by an ambulance on the other side of the border [22].

## 7. Challenges Faced in Provision of Supplies for Newborn Screening

Shortages of the supply of protective equipment were notable at the start of the pandemic. Due to transport restrictions and supply chain issues, important materials for NBS (e.g., reagents, testing kits, pipette tips, and other laboratory consumables) were also in short supply [16,24]. Moreover, access to technical assistance for the maintenance of NBS laboratory equipment was sometimes not available [17]. A reduced supply of pipette tips led to considering methods for their cleaning and reuse [15]. Supplies were also sometimes shared between NBS laboratories, and suppliers were directly contacted to arrange extra shipments [10,16].

## 8. Challenges Faced with Laboratory Evaluation of Newborn Screening Samples

In the early phase of the pandemic, the transmission of COVID-19 from blood spots to laboratory workers was questioned, and protocols to reduce transmission were introduced [15]. The general principles of careful sample handling continued to be applied in the analysis of NBS specimens. The use of protective equipment and hand hygiene were employed as usual. If it was suspected that aerosols or droplets could be produced during the process, working inside a biological safety cabinet or chemical fume hood was recommended, and the use of protective equipment, for example, N95, FFP2, and KN95 masks, was implemented [7,25,26]. Standard decontamination procedures for work surfaces and rooms were recommended using registered disinfectants [7,27]. Some laboratories recommend UV light or ozone generators, but the EPA did not verify their use [27]. It was suggested that, following analysis, specimens should be sealed immediately, and if suspicion of COVID-19 infection existed, it was recommended that the sample should be promptly disinfected or autoclaved [25].

The Colorado newborn screening program published guidance for performing NBS under emergency conditions to ensure timely testing of critical NBS samples. Time-critical disorder assays were given priority for testing (for example, first screen samples for MS/MS: GALT, TSH, and 17OHP). Cystic fibrosis was not categorized as a time-critical disorder, but this resulted in an increased turnaround time. Other conditions, such as SCID, SMA, CF DNA, and biotinidase deficiency, as well as hemoglobin testing, were not categorized as time-critical and were analyzed at least weekly on different time schedules, depending on daily workload. Repeated samples were given a lower priority; however, abnormal initial tests subject to a new sample testing remained a high priority [28].

Scanning software for DBS cards with built-in intelligent character recognition was planned to be used in some centers to automatically read the DBS cards’ information and import the data directly to the laboratory information management system [6]. Novel information laboratory systems were implemented to enable remote working, but reduced IT support sometimes interfered with these plans [15]. Additional funding was required to utilize these new approaches [15].

## 9. Challenges Faced in Communication of the Team Members and Participants of Newborn Screening

In person, congresses and meetings were limited, cancelled or postponed because of COVID-19 to minimize the transmission of the disease. Abnormal NBS results sometimes require a new sample test collection and further evaluation and services, and occasionally it was difficult to schedule these visits. Providers also found it difficult to connect families to physician support with lengthy telephone waits, some lasting up to 40 min [7]. Some parents feared the exposure of their newborns to a COVID-19 infection from a face-to-face encounter [16].

To help overcome these difficulties, laboratory staff in Iowa directly contacted the families to arrange confirmatory testing. Online educational packages were created to assist primary care physicians when providing information for families or arranging appropriate confirmatory testing. Key emergency contacts to handle urgent results needed to be identified in some instances [7].

In May 2020, the Association of Public Health Laboratories initiated NBS webinars for information exchange and added them to their website (The Newborn Screening Technical assistance and Evaluation Program) [7]. They also added resources to their webpage and provided examples of problems encountered along with suggested solutions [7]. Communication by phone, email or instant messaging proved helpful [6,29]. To maintain good information governance when working from home, an upgrade of VPN servers was needed in some cases [6].

Telemedicine/web-based consultations were used to replace face-to-face visits in many situations. Evidence suggested that they could be used as a safe and effective method of communicating abnormal NBS results [30,31]. In the study by Gold JI and coworkers, an increased use of telemedicine during the pandemic was observed without a negative impact on the experience for the family, as reflected in their likelihood to recommend the use of providers to a friend. The inability to perform a physical examination was mentioned as a disadvantage, but not all participants agreed [31]. 

Telemedicine could also be provided by nurse practitioners, certified nurse midwives, clinical psychologists, clinical social workers, registered dietitians, and others, but in some situations, this was subject to state regulation [32]. Telemedicine also played an important role in the further management of patients with inherited metabolic disorders to help avoid COVID-19 infection while continuing regular care [1,33].

The limitations of telemedicine were explained to the caregivers before the visit [22]. The language barriers are more noticeable in telemedicine compared with face-face visits; for example, the use of body language is not feasible. Furthermore, the accessibility of interpreters for telemedicine was problematic during the COVID-19 pandemic [34]. In Washington, the hospital system rapidly improved access through virtual interpreter services. An online folder, accessible to the entire division, with information, standard operating procedures, workarounds, division-wide contacts list, and documented rapid solutions was created [35].

## 10. Challenges Faced in the Management of Abnormal Newborn Screening Results

Idiosyncratic factors (prematurity, use of antenatal steroids, intrauterine stress, and maternal infection) also could affect the interpretation of NBS results. However, reports of COVID-19 cases where idiosyncratic factors affected NBS results are rare, despite the large number of COVID-19 pregnancies. Mak et al. describe the case of a newborn of a COVID-19-positive mother with a false positive screening result for congenital adrenal hyperplasia [36]. To ensure that every baby was screened at least once, acceptance criteria were relaxed, and reports were produced on blood spots of lesser quality [6,15]. The pandemic required NBS staff to make greater use of electronic means of reporting to replace letters, and this required additional input from IT to ensure that robust and secure systems were in place [15].

## 11. Challenges Faced at the Hospitals and Maternity Wards to Perform Newborn Screening

The need for increased numbers of hospital beds and related space requirement to treat COVID-19 cases reduced the space to perform NBS [22]. An increased hesitancy to return to the clinic for taking new dried blood spots tests was observed in families with newborns [15]. Strict enforcement of distancing and mask protocols were established for clinical visits to ensure the safety of the staff and patients [22]. 

## 12. Challenges Faced in Research and Implementation of Novel Screening Methods for Newborn Screening

The COVID-19 pandemic had an impact on pediatric research, but at the same time, it provided an opportunity to explore novel ways of planning and delivering services, including NBS [37]. Before including a new condition into existing NBS programs, research and evaluation were required, and practical aspects had to be adapted to comply with the circumstances created by the COVID-19 pandemic. In New York, for instance, while pre COVID-19, a Duchenne muscular dystrophy pilot was usually undertaken, the in-person informed consent protocol had to be changed to a remote recruitment (by phone or online). Prior to the pandemic, parents were visited within 72 h after birth and were given a study brochure and shown a three-minute video about the study. Following the onset of the COVID-19 pandemic in March 2020, this was modified to permit verbal consent by phone, or an electronic or paper consent via a return envelope [9].

After gaining greater knowledge about COVID-19, restrictions were relaxed, and a hybrid (in-person and remote recruitment) approach was used. Recruitment by phone was used to contact those with an early discharge and when results of the routine NBS panel were available. Formal consent was then subsequently confirmed by e-mail or by post. Remote data entry was also used [16]. All this had to be managed and recorded in the information systems. This required additional fields to be created to collect contact information and track phone calls and e-mails to and from participants, and a daily list of consented babies was sent to the laboratory [9]. A hybrid approach could also be used for those who were discharged at weekends or holidays [9]. In England, six NBS laboratories began an evaluation for SCID screening, and the discussions to begin screening were conducted virtually so that the SCID program could commence in September 2021 [19].

## 13. Conclusions

The COVID-19 pandemic and public health measures had differing effects in different countries or social groups. Perhaps, as might be anticipated, a greater impact was sometimes observed among those who were socioeconomically disadvantaged, and this extended to an effect on NBS services [10,38].

Performing NBS is particularly important to ensure the early detection and treatment of children with rare and inherited diseases. This was also recognized by some governments; for example, in Wisconsin, NBS was deemed an essential service, protecting staff from being transferred to other duties, including COVID-19 testing [16].

A lot can already be learned from routine NBS practices and from actions during previous natural disasters (hurricanes), pandemics, and other times of crisis. Some countries were already performing some of the suggested actions during the pandemic as part of the implemented NBS actions routinely for many years, such as the 6-day working week, as well as shifts divided into two daily shifts being performed in Germany after the newborn screening directive entered into force in 2005 [39]. Moreover, some countries have already implemented taking samples for NBS in an earlier time frame from the age of 24 h on (Spain, Montenegro, and Kazakhstan) or from the age of 32 or 36 h onwards [4].

**Figure 2:**
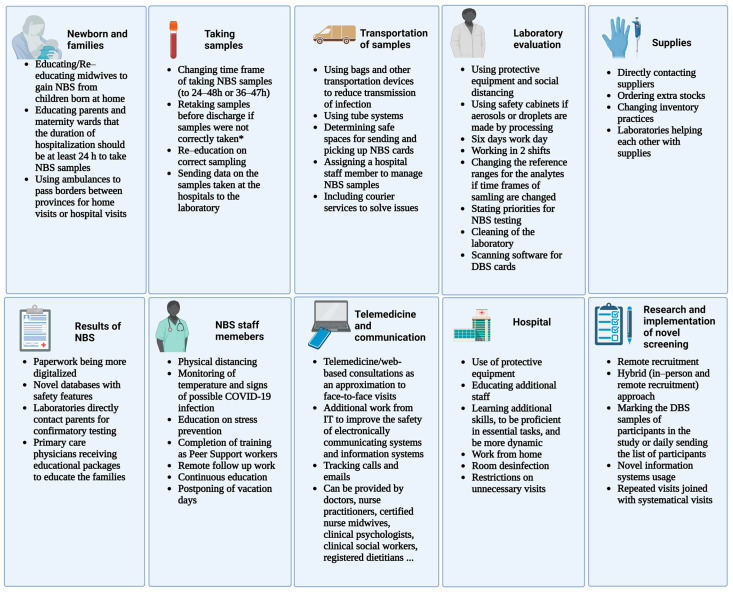
A summary of contingency plans proposed for performing newborn screening during the COVID-19 pandemic. Legend: NBS—newborns screening, DBS—dried blood spot, and IT—information technology. * The samples should be retaken if they are taken before the given time frame or if taken incorrectly, and this is already seen at the hospital (created with BioRender.com (BioRender, Toronto, ON, Canada)).

During the COVID-19 pandemic, a lot could additionally be learned to enable the continuation of NBS in a time of crisis. A summary of contingency plans is presented in Figure 2. Personal protective equipment, hygiene, and social distancing were the first steps to prevent additional staff shortages. Work from home and new time schedules were implemented to reduce the number of staff at the locations and prevent the spread of the virus. The stress and workload were enormous. Therefore, preventive strategies are essential to reduce burned-out persons [10,15,16]. Because of shortened hospitalizations of newborns, novel protocols with novel reference ranges for the analysts had to be made in some centers [6,7,16]. The handling of the specimens in the laboratory mostly had to be done, as it was known for other infectious diseases, but a shortage of protective equipment was seen early on [7,24,25,26]. NBS had to interchange equipment between them or directly contact the suppliers [10,16]. Moreover, couriers had to be contacted directly to sustain delivery of specimens in an acceptable time range [16]. Telemedicine/web-based consultations were used more often during the COVID-19 pandemic, and evidence suggests that they could be used as a safe and effective method of communication between NBS staff and also to communicate NBS results continuously, not just during a time of crisis [30,31]. IT support was needed to make work from home possible and safe and to make telehealth possible [6].

Additionally, the adoption of electronic messaging using standard codes could allow faster result reporting and improve patient follow-up and continuity of care, as well as exchange and aggregate NBS results from different centers [40]. Before a time of crisis, constant improvement of assays and development of second-tier strategies to investigate altered biomarker levels in primary screening results is also important. Consequently, a reduction in the number of false positive results and the number of revisits is observed [41].

Most of the centers did not have contingency plans for performing NBS in the case of a pandemic [10]. In some places, new service continuity plans were introduced within the first week of the Pandemic having been declared and this emphasized the importance of careful planning and preparedness to ensure the success and sustainability of NBS services [6,16]. Contingency plans help to ensure availability of critical resources, not only in a pandemic but also in service interruptions caused by natural disasters and the inability to provide testing materials etc. [42]. Still a lot of countries did not have contingency plans to maintain NBS during a time of crisis in 2021 [10].

It appears that, to maximize the performance of NBS during a time of crisis, national and international collaborations are important [10] and it is also important to include other neonatal screening methods (hearing, hip dysplasia and others), which were not covered in this article, in the contingency plans.

## Data Availability

Not applicable.
